# An Automated and Continuous Plant Weight Measurement System for Plant Factory

**DOI:** 10.3389/fpls.2016.00392

**Published:** 2016-03-31

**Authors:** Wei-Tai Chen, Yu-Hui F. Yeh, Ting-Yu Liu, Ta-Te Lin

**Affiliations:** Department of Bio-Industrial Mechatronics Engineering, National Taiwan UniversityTaipei, Taiwan

**Keywords:** growth curve modeling, fresh weight, hydroponics, vegetables, plant growth, load cell

## Abstract

In plant factories, plants are usually cultivated in nutrient solution under a controllable environment. Plant quality and growth are closely monitored and precisely controlled. For plant growth evaluation, plant weight is an important and commonly used indicator. Traditional plant weight measurements are destructive and laborious. In order to measure and record the plant weight during plant growth, an automated measurement system was designed and developed herein. The weight measurement system comprises a weight measurement device and an imaging system. The weight measurement device consists of a top disk, a bottom disk, a plant holder and a load cell. The load cell with a resolution of 0.1 g converts the plant weight on the plant holder disk to an analog electrical signal for a precise measurement. The top disk and bottom disk are designed to be durable for different plant sizes, so plant weight can be measured continuously throughout the whole growth period, without hindering plant growth. The results show that plant weights measured by the weight measurement device are highly correlated with the weights estimated by the stereo-vision imaging system; hence, plant weight can be measured by either method. The weight growth of selected vegetables growing in the National Taiwan University plant factory were monitored and measured using our automated plant growth weight measurement system. The experimental results demonstrate the functionality, stability and durability of this system. The information gathered by this weight system can be valuable and beneficial for hydroponic plants monitoring research and agricultural research applications.

## Introduction

A plant factory is an indoor cultivation space where the growing environment of plants is carefully controlled, including factors such as light, temperature, carbon dioxide, and nutrient solution. Hydroponics is a cultivation method commonly used in plant factories, where plants are grown without soil, but rather with nutrient solution. Compared with soil cultivation, hydroponics has the advantages of conservation of water and nutrients, as well as more complete control of the environmental factors affecting plant growth (Jones, 2004). A plant factory is intended to achieve the stable and optimized production of plants by controlling the growing environment. Therefore, growing plants in a plant factory also becomes a control problem in engineering. Plant growth is responsive to the environmental parameters. Therefore, by manipulating the growing environment, plant growth can be controlled as expected. There already exist different sensors for measuring environment parameters, such as photometer, thermometer, humidity sensor, and electrolyte analyzer. In order to measure plant responses to the environment, various approaches have been developed to quantify plant features such as leaf area and plant weight as affected by different growing conditions.

To quantify plant growth, plant weight is an important feature. However, the traditional method for plant weight measurement, which measures plant weights manually by picking plants up and measuring weight using an electronic balance, is not only destructive but also laborious. Sase et al. (1988) measured the weight and leaf area of lettuce under different sources of light. The results showed no significant difference in fresh weight, dry weight and plant area between lettuce grown under high-pressure sodium lamps and metal halide lamps. Therefore, the research indicated that spectral power radiation from lamps has no influence on fresh or dry weight on a specific range of photosynthetic photon flux (Sase et al., 1988). Van Henten and Bontsema (1995) showed a linear relation between the leaf area and the dry weight of lettuce through examining the results from image processing methods and destructive plant weight measurements; they indicated the possibility of a non-destructive plant growth measurement method using the plant leaf area to estimate the plant dry weight. As there are too many input environmental parameters to be adjusted in a plant factory, the traditional weight measurement method can hardly be applied due to its time and labor costs. Therefore, an automatic plant weight measurement instrument which can measure plant weight continuously during plant growing period is needed for plant factory.

Some automated plant weight measurement instruments have been developed. Takaichi et al. (1996) developed an instrument which automatically measures tomato plant fresh weight using two electronic balances. A tomato plant growing in a pot with nutrient solution was suspended. The total weight of the pot was measured using one electronic balance, while the weight of nutrient solution was measured using the other electronic balance. This instrument automatically and continuously realizes plant weight measurement for hydroponics (Takaichi et al., 1996). However, the instrument can only be applied in the laboratory, as it requires two electronic balances to measure a single plant’s weight. Baas and Slootweg (2004) developed an on-line plant monitoring equipment for horticulture, which measures the weight of a group of Gerbera using multiple load cells. A group of Gerbera was growing on the Rockwool slab. The weight system mounted with load cells was put under the Rockwool slab to automatically measure the total weight of plants (Baas and Slootweg, 2004). This instrument achieves automated plant weight measurements in real cultivational space; however, it can only measure the total weight of a group of Gerbera, not a single plant’s weight. In order to build a plant growth monitoring model which is responsive to environment changes, the environment parameters must stay consistent for all growing plants, which is difficult to achieve. Therefore, an instrument made for single plant measurement is still desired. Helmer et al. (2005) developed a system called CropAssist, which can automatically measure plant weight and transpiration for vine crops, such as tomatoes and cucumbers, using pairs of load cells and a trough system. CropAssist is a commercial system which can measure plant weights and transpiration rates of single or multiple plants using two load cells. The weights of vine plants were measured by the upper load cell which suspends the vine plants. The transpiration rates were estimated by the lower load cell which measures weight changes of growing media (Helmer et al., 2005). However, this system can only work for vine crops and not suitable for leafy vegetables, because it is not convenient to hang leafy vegetables and leafs touching the ground can introduce notable errors to CropAssist system. Furthermore, considering space efficiency, CropAssist system is too bulky to use in plant factories.

In the present research, an automated and continuous plant weight monitoring system is proposed. This system includes two main parts: an automated plant weight measurement instrument and a stereo-vision imaging system. Since the plant weight measurement instrument consists of two acrylic plates and a load cell, it is easy to build and apply in practice. Hydroponic plants were grown on our instrument from budding to harvesting. During the plant growth period, the weight of the plants was measured using this weight measurement instrument, and compared with imaging features captured by the cameras in the stereo-vision imaging system. These instruments were applied to measure weight growth of Boston lettuce and coral lettuce cultivated in the National Taiwan University plant factory. Also, destructive measurements were carried out to validate the measurement accuracy of this system. Finally, continuous plant weights were measured by both this weight measurement instrument and the imaging system.

## Materials and Methods

### Instrument Design

**FIGURE 1:**
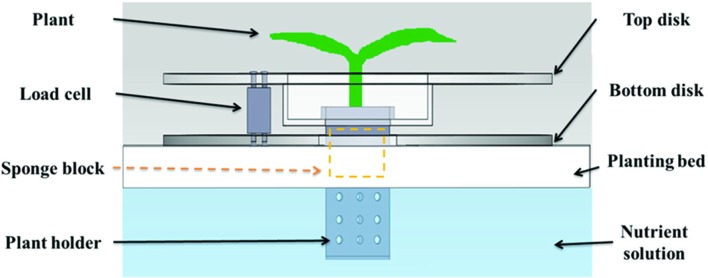
**The design of weight measurement instrument**.

The sensor used to measure plant weight in our weight measurement instrument is a load cell. A load cell is a transducer which converts a force signal into an electric signal. In this research, LDB-2 kg load cells (Esense Scientific Ltd., Taiwan) were used, with a measuring resolution of 0.1 g, and capability to measure weight from 0 to 2 kg. Our automated weight measurement instrument has four components: a top disk, a bottom disk, a plant holder and a load cell. As **Figure 1** illustrates, the plant is fixed on the sponge block, which is clinched by the plant holder. The plant holder is hung on the top disk, which is also fixed with one top end of the load cell. The bottom disk is fixed on the bottom of the other end of the load cell. Since the load cell connects the top disk and the bottom disk, and the bottom disk is placed on the planting bed for support, the total weight on the top disk creates a downward force onto the load cell which is then converted to an analog electric signal. The load cell is connected to a amplifier and conditioner module (Model: JS300, Jihsense Ltd., Taiwan) which amplifies and converts the electric signal to a proportional weight value. This weight signal is then sent to the computer via RS232 connector. The plant to be measured is fixed and grown on the plant holder. Therefore, as the plant grows, the weight change can be measured continuously by the load cell. The plant weight measured by the instrument is only the plant shoot, since the plant root is immersed in the nutrient solution and the weight is canceled out by the buoyant force. The top disk and bottom disk both have an opening hole in the middle, so the shoot part can grow upward through the top disk, and the root can grow downward and immerse in a nutrient solution of the hydroponics system. The top disk is designed in a bowl shape to lower the plant holder, so the root can immerse into the nutrient solution even during the budding period. The plant holder is designed as an enclosure with holes to avoid roots tangling with other plants, and to ensure circulation of nutrient solution.

### System Setup and Cultivation Environment

All experiments were carried out in the National Taiwan University plant factory. This pilot plant factory is an indoor hydroponics environment. The environmental conditions, including light, temperature and nutrient solution, are artificial, and controllable. The light source is provided by the fluorescent light instead of sunlight, so the light intensity and light period is controllable. The average light intensity on the planting bed is 169 μmole/s.m^2^. The temperature in the planting bed is stably controlled and monitored by an air conditioner and a temperature sensor. The day and night temperature were set at 23 and 19°C, respectively, with 16 h of light per day. The light period starts at 8:00 and the dark period starts at 0:00. The typical CO_2_ concentration in the plant factory ranges from 300 to 500 ppm in 1 day timeframe. The nutrient solution is an A–B bottle mixed solution containing Magnesium carbonate, Calcium carbonate, Potassium carbonate, chelated iron, boric acid, Sodium molybdate, Copper sulfate, Manganese sulfate, Potassium chloride, Potassium phosphate, and Zinc sulfate, provided by the Department of Horticulture and Landscape Architecture of National Taiwan University.

**FIGURE 2:**
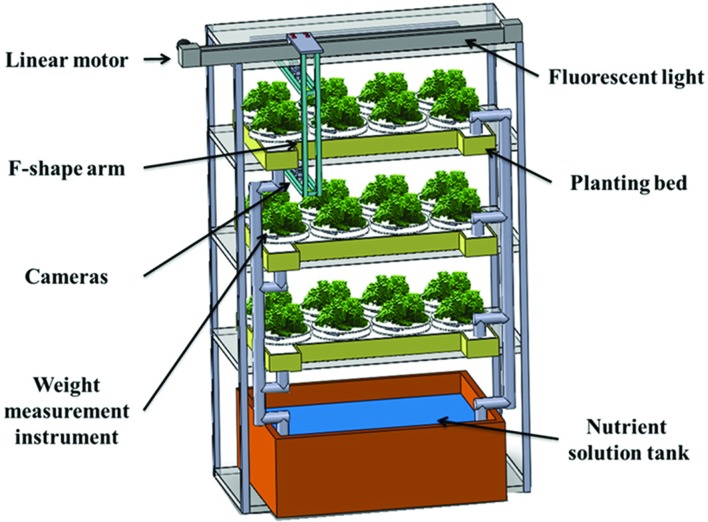
**System schematic diagram in a plant factory**.

**FIGURE 3:**
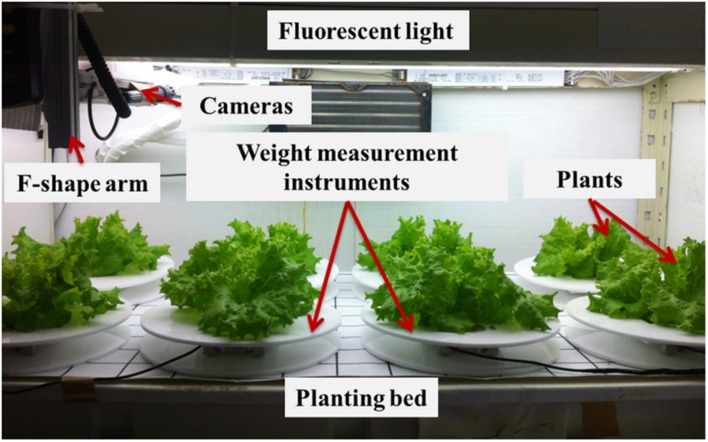
**Real scene in National Taiwan University plant factory**.

This weight monitoring system was set up in the planting room of the National Taiwan University plant factory, as shown in **Figures 2** and **3** shows the real scene of one planting bed. There are multiple vertically arranged planting beds in one growing shelf stand. For each planting bed, there are T5 fluorescent lights to provide illumination, and circulating essential nutrient solution for the plants. The nutrient solution is circulated with the nutrient tank at the bottom of the growing shelf stand to ensure a stable supply of the nutrient. There are eight holes uniformly distributed on the 110 cm × 50 cm planting bed, with one weight measuring instrument in each hole, so eight plants can be monitored concurrently in one planting bed. The opening of the weight measurement instrument bottom disk is aligned with the hole of the planting bed to ensure that the root can immerse in nutrient solution. The plant weight’s analog electric signal provided by the load cell is transferred to a computer via an analog-digital converter for recording purpose. A user interface program was developed to record and display the plant weight in real time. In order to compare plant weight measured by load cell with those measured by the stereo-vision method, an imaging system is used to simultaneously measure geometric features of plants. Cameras mounted on the F-shape arm are driven by a motor to take images above the same plant bed. Geometric features of measured plants are calculated based on the stereo-vision approach. The details of the imaging system and algorithms are proposed and provided in a previous paper (Yeh et al., 2014).

In this research, two species of lettuce, Boston lettuce (Known-you seed Co., Ltd. LS-047) and coral lettuce (Known-you seed Co., Ltd. LS-005) were grown and measured using this weight monitoring system and the imaging system. The plants were grown in nursery pots for 14 days for germination. After the roots of the plants were long enough to immerse in the nutrient solution, the plants were transplanted to the weight measurement instrument’s plant holders to grow on the planting bed. In each experiment, weight measurement instruments with plants in the plant holder were arranged carefully on the planting bed. The nutrient solution was circulating between the planting beds and storage tank with a pump to ensure that the nutrition is uniform during the experiments.

## Results

### Weight Measurement Validation

**FIGURE 4:**
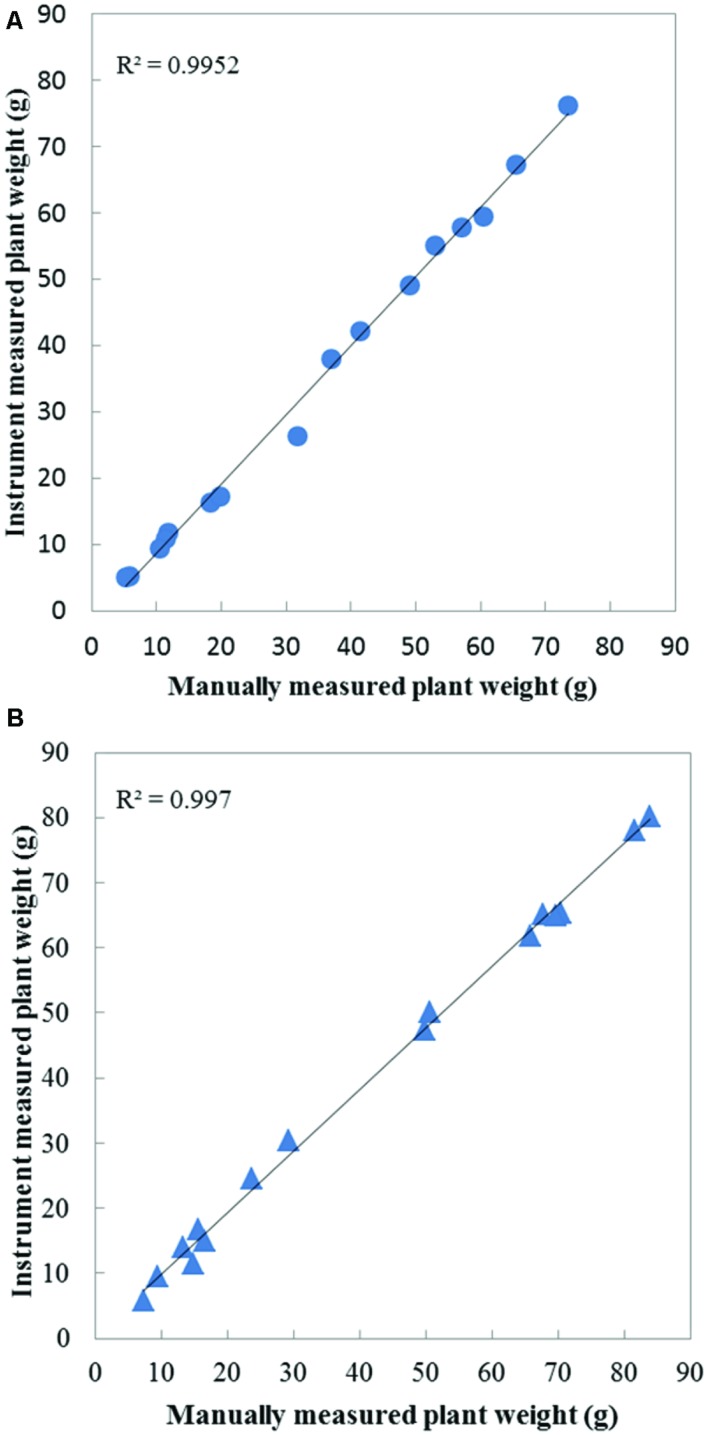
**Weight measurement accuracy validation for **(A)** Boston lettuces (circle) and **(B)** coral lettuces (triangle)**.

In order to validate the accuracy of our weight measurement instrument, 16 Boston lettuces and 16 coral lettuces which were grown were picked up and measured destructively using an electronic balance across the 14 day growing period. **Figure 4** shows the results of plant weight measurement validation of the Boston and coral lettuces. The vertical axis represents the last weight measurement recorded by the instrument before destructive measurement. The horizontal axis is the weight of the plant shoot part measured by an electronic balance, which is regarded as the ground truth. Since the weight of the plant root was canceled out by the buoyant force, as the density of roots is very close to nutrient solution, an average density of 0.98 g/cm^3^ for lettuces and 0.99 g/cm^3^ for the nutrient solution. The plant weight measured using this instrument is close to the shoot part only. The average error between the instrument’s weight measurement and electronic balance’s measurement for the Boston lettuce is 5.66, and 6.99% for the coral lettuce.

Since this weight measurement instrument is designed not to affect plant growth, one round of cultivation experiment was carried out to confirm this design principle. In this validation experiment, 16 Boston lettuces were monitored for 24 days, eight of them were grown with the weight measurement instrument on the planting bed and the other eight were grown without the instrument. At the end of the experiment, the plant weights were measured by the electronic balance; eight Boston lettuces grown on our instrument had an average weight of 202.1 g with a standard deviation of 19.2 g. The other eight Boston lettuces grown solely on a planting bed had an average weight of 201.3 g with a standard deviation of 26.4 g. It is clear that the plant weights with and without the weight measurement instrument are close and the appearance is alike, which proves that the instrument does not affect the plant growth.

### Continuous Weight Measurement

**FIGURE 5:**
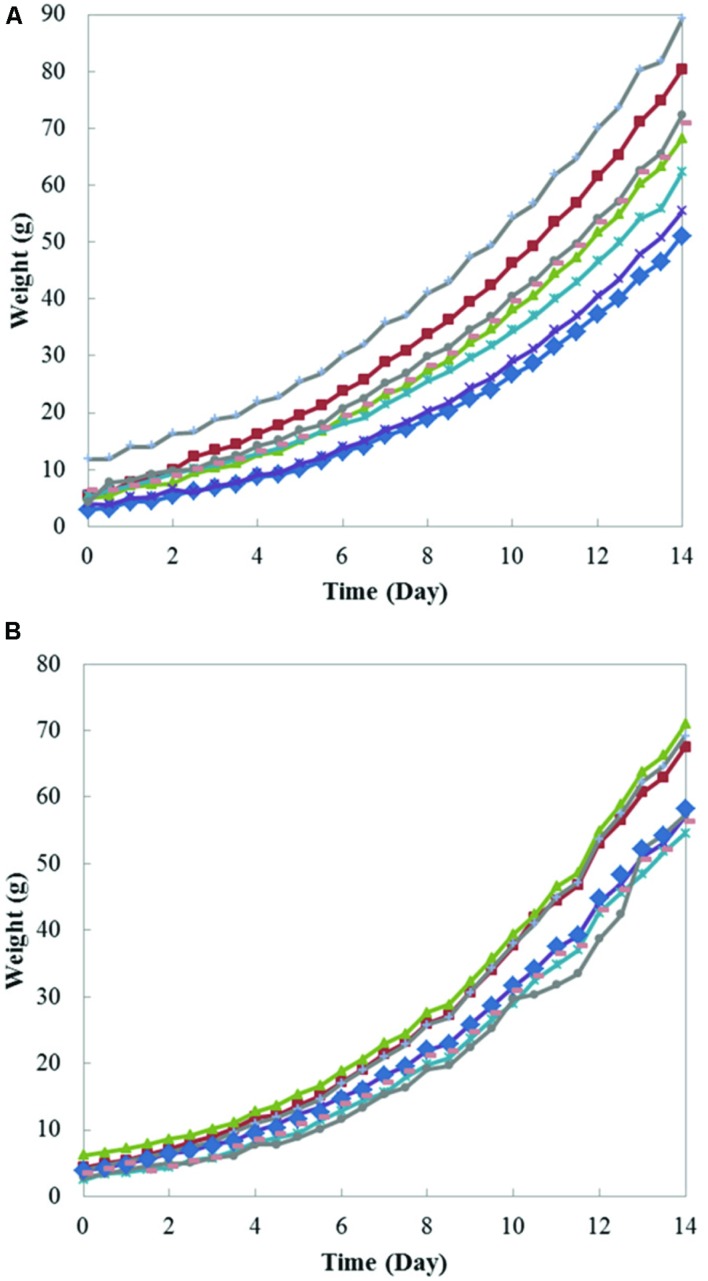
**Plant weight growth curves of **(A)** eight Boston lettuces and **(B)** eight coral lettuces**.

This weight measurement instrument measures individual plant’s weight non-destructively and continuously in a hydroculture environment. Therefore, the growth of individual plants during the growth period can be monitored and recorded using this instrument. **Figure 5** shows the weight growth curves of eight Boston lettuces and eight coral lettuces measured. Each point on the figure is the average weight measured in that particular hour. It can be seen that both Boston lettuce and coral lettuce show similar weight growing pattern. At the beginning, the weight changes were relatively small, since the plants were just transplanted onto the planting bed. This is the root growing period. The weight changed faster as the plants grew larger.

**FIGURE 6:**
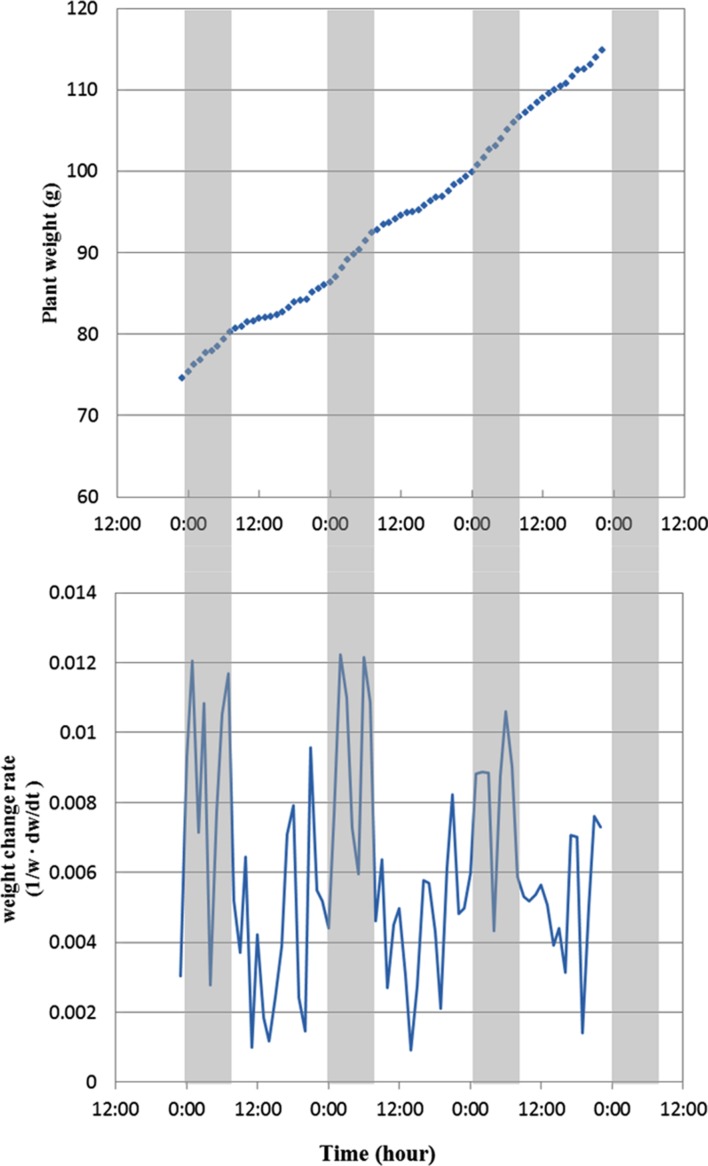
**Diurnal change of plant weight **(top)** and weight change rate **(bottom)** from day 15 to day 18 of the growing period. The gray area indicates the dark period, and white area is light period**. The weight change rates are calculated as the weight difference between previous and current hour over current hour weight.

Based on plant growth curve, further information can be extracted and analyzed. **Figure 6** shows the diurnal change of a Boston lettuce in weight from day 15 to day 18 of the growing period. The weight change rates are calculated as the weight difference between previous and current hour over current hour weight. The result shows a diurnal pattern of plant weight change rates. The change rates are all positive but smaller at the beginning of the light period and larger at the end of light period. The plant weight change rates remain large during the dark period. This phenomenon coincides with other researches which show plants grow rhythmically in association with light accessibility (Normann et al., 2007; Nozue et al., 2007). This result shows that the weight measurement precision is sufficient to detect plant growth rhythm due to light accessibility; thus, the system provides another useful function for plant physiological research.

### Plant Weight Measurement Instrument and Imaging Measurement System

**FIGURE 7:**
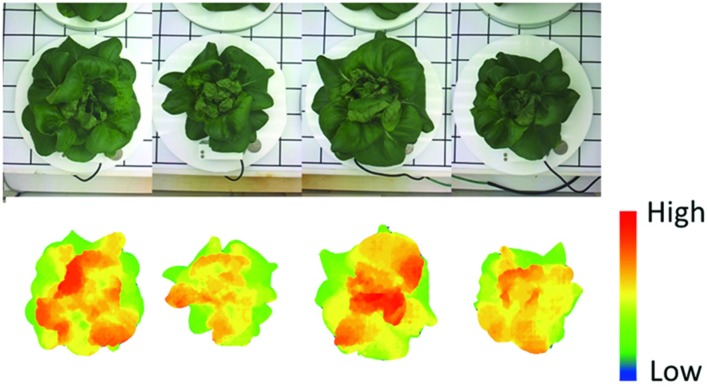
**The (top) is the raw image of Boston lettuces and the **(bottom)** is the corresponding pseudo-color depth image calculated by stereo-vision algorithm**.

**FIGURE 8:**
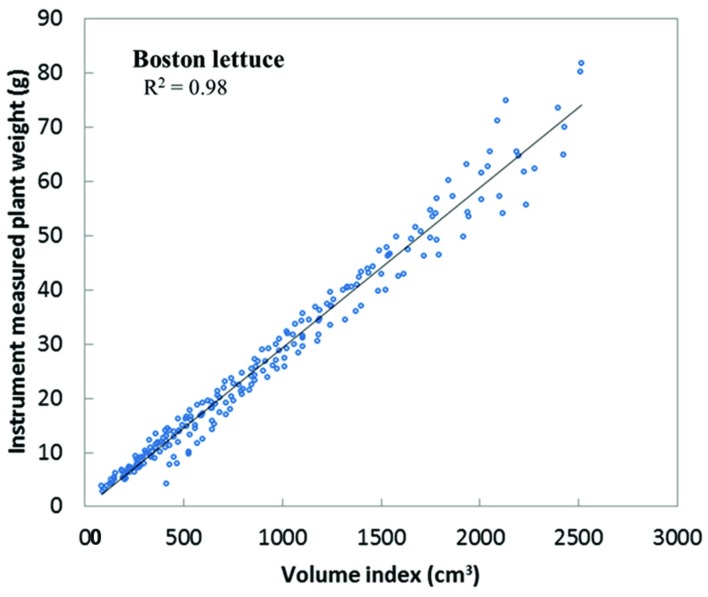
**Linear model built between plant weight and plant volume index**.

**FIGURE 9:**
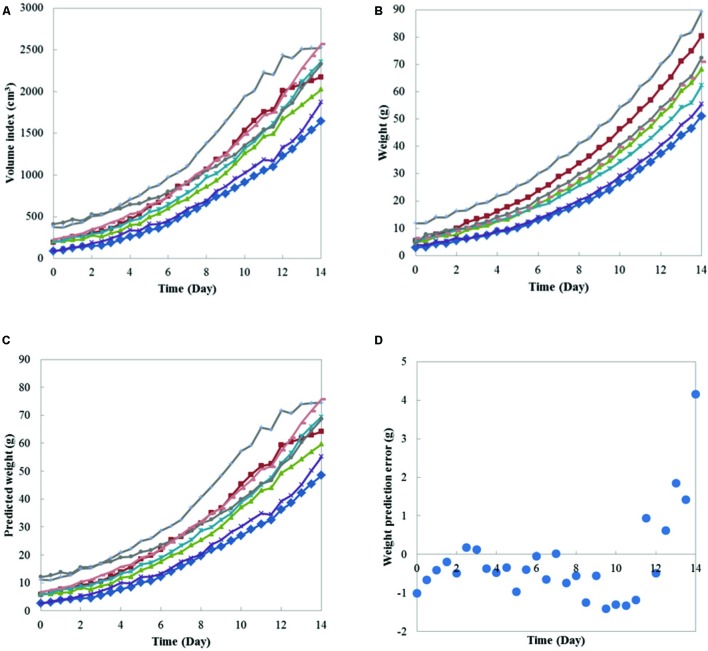
**Growth curves of eight Boston lettuces. (A)** volume index measured by the imaging system; **(B)** weight measured by the weight measurement instrument; **(C)** weight predicted from the volume index measured; and **(D)** the prediction error over the growth period.

In this monitoring system, there is another method to measure plant weight, the stereo-vision imaging system. The weight and imaging feature of plants obtained show high correlation across plant growing periods. Eight Boston lettuces were grown and measured by both the weight measurement instrument and the stereo-vision imaging system. By applying stereo-vision techniques, the plant volumes can be calculated from captured plant images, as shown in **Figure 7**. In **Figure 7**, the top is the raw image of Boston lettuces and the bottom is the corresponding pseudo-color depth image calculated by stereo-vision algorithm. The plant volume index can be further derived based on the depth image of the plants. Since both weight and vision measurement were taken continuously during the plant growth period, the relationship between plant weight and plant volume index can be found. **Figure 8** shows that a linear model existed between plant weight and plant volume index based on this round of experiment, and a high R-square of 0.98 was obtained. Based on this linear model, plant weights can then be estimated based on the volume index data, as shown in **Figure 9**. As the weight and volume index of plants have a linear relationship between them, the growth curves of weight estimated by the volume index would have the same pattern as the volume index ones as shown in **Figures 9A,C**. The errors between predicted weight and measured weight across the growth period are plotted in **Figure 9D**. It is clear that the errors are within 1.5 g for the first 10 days and relatively larger at the later part of the growth period. This is understandable: as the plant grows bigger, the error of volume estimation from the image becomes greater due to void space in the plant volume. Hence, the weight estimated by the volume index is affected by this cumulative error. However, this estimation error is still much below 1% of the actual weight. The result shows the possibility and capability of estimating plant weight from the volume measurement using a simple imaging system.

## Discussions

For both the Boston lettuce and coral lettuce, compared with the traditional destructive measurement method, the weight measured by our instrument has minor error; however, automated and non-destructive measurement is achieved. Furthermore, the result shows that the weight measurement instrument can be applied to hydroponic leafy plants which can be of different geometric shapes. In our weight measurement instrument design, the top disk is used to support hanging down leaf. For different plant species, the diameter of top disk can to be changed accordingly in order to support all hanging down leaf of plant. Also, the hole on the top disk and bottom disk can be modified to the size for stems and roots to fit.

From the results presented, the weights between plants disperse due to the spatial non-uniformity of light intensity, as well as temperature variation to each plant. The plants grew faster with the higher light intensity supplied. The difference between Boston lettuce and coral lettuce can also be pointed out via comparing two figures in **Figure 5**. The coral lettuces generally have lighter weights than the Boston ones with smaller variance in weight between plants. This result shows that the instrument can measure plant weight continuously during the growth period. In a plant factory, small variation of plant weight is to be expected. Thus, besides the final plant weight, the variability of plant weight is an indicator for the growers to adjust their operations and environmental parameters. Using the current measurement system, a couple of plant growth curves can be acquired spontaneously and concurrently. Compared with traditional destructive measurements, which require 100s of manual measurements to get same amount of data, our instrument can reduce labor and save time for experiments. Hence, this continuous weight measurement system is an efficient tool for plant factories to obtain optimum growing condition of crops.

Comparing the plant weight instrument and the imaging system, the automated weight measurement system is required to calibrate the imaging system. Hence, the imaging system cannot be applied solely at the beginning. For different growing environment and different species of crop, the calibration needs to be retained again. Therefore, both the automated imaging system and weight measurement system are needed at the set up phase of the system. The automated weight measurement system is more compact and portable to fit different hydroponic environment. Furthermore, the automated weight instrument provides direct measurements, no calibration required. On the other hand, the main advantage of using imaging information is that the weights of multiple plants only need to be measured once after the calibration model between plant weight and volume index is determined. The imaging measurement system is equipped with moving cameras, so only a few cameras are required to measure the plant weight of numerous plants by converting the volume index to estimated plant weight using the calibration model. It is therefore worth noting that the weight measurement instrument exhibits another important functionality to efficiently build a calibration model between image features (projected leaf area, volume index, etc.) and the plant weight for specific plants. This kind of calibration model was usually difficult to build and could only be obtained using destructive method.

Nevertheless, in the future study, we aim to build an instrument based on this design to measure the weight of whole plant, including the shoot weight and the root weight separately. The hole on the disk is large enough for the stem of lettuce to grow throughout the entire growth period. In addition, the hole do not affect the stand stability of lettuces, since the plant is not supported by the hole of disk but fixed on the sponge block, which is clamped by the plant holder. For root weight measurement, it is possible by draining out the nutrient solution to measure the whole plant weight and thus to obtain the root weight.

## Conclusion

In this research, an automated, non-destructive plant weight measurement system for hydroponic plants is developed, which includes a self-developed weight measurement instrument and a stereo-vision imaging system. The weight measurement instrument made of a load cell and acrylic plates is able to continuously measure the weight of the plant shoot part not specific to any hydroponic plant. The accuracy of the instrument is validated by comparing instrument results with electronic balance readings with an average of 6% measurement error. The experiment’s results also show that plant growth is not affected by the attachment of the instrument to the plant. Successful measurements of growth curves of Boston lettuce and coral lettuce in a plant factory were demonstrated. Furthermore, the diurnal pattern of plant weight change rate in light and dark periods can be observed through this system. There is another approach to retrieve plant weight during plant growth period, which is to use plant weight and imaging measurement to find a calibration model between them. Once the calibration model is found, only the image information of plants is needed to estimate plant weight during the plant growth period. Below 1% error in weight estimation across the whole growth period is demonstrated. This research shows the functionality of this automated weight measurement system and the possibility of future contribution in practical applications for plant factories, as well as for various plant physiological researches.

## Author Contributions

W-TC, T-TL: conception, design, and implementation of the research; W-TC, Y-HY, T-YL, T-TL: acquisition and analysis of the data; WC, T-TL: interpretation of data; WC, Y-HY, T-TL: drafting the manuscript.

## Conflict of Interest Statement

The authors declare that the research was conducted in the absence of any commercial or financial relationships that could be construed as a potential conflict of interest.
